# Mechanistic insights into polycystic ovary syndrome and periodontal disease: a systematic scoping review of inflammatory, oxidative stress, hormonal–metabolic, and microbial pathways

**DOI:** 10.1007/s44445-026-00254-9

**Published:** 2026-10-26

**Authors:** Mustafa Nassar, Yasmina Zatar, Nino Sharikadze, Tamar Aladashvili, Eka Kokhreidze

**Affiliations:** 1 European University, Tbilisi, Georgia; 2 National Center for Tuberculosis and Lung Disease, Tbilisi, Georgia

**Keywords:** Polycystic ovary syndrome, Periodontal disease, Systemic inflammation, Oral-systemic health, Reproductive health, Immunoendocrine dysfunction

## Abstract

To identify critical evidence gaps, this scoping review mapped the extent and nature of published research exploring the biological links between polycystic ovary syndrome (PCOS) and periodontal disease. Following PRISMA-ScR guidelines and a pre-registered protocol (OSF: https://doi.org/10.17605/OSF.IO/WE3XU) a systematic search of PubMed, Scopus, EBSCO, and Google Scholar identified original human studies assessing clinical periodontal parameters and mechanistic biomarkers in PCOS patients. Screening and data extraction were conducted independently by two reviewers. Thirty-three studies met the inclusion criteria. Mapping identified a synergistic pathophysiological network across four domains. The evidence reveals an elevated systemic inflammatory baseline marked by hyper-activated endothelial markers (Endocan) alongside elevated levels of Tumor Necrosis Factor-alpha (TNF-alpha) and Interleukin-6 (IL-6), which appears to lower the threshold for local tissue destruction. This is coupled with a compromised antioxidant defense—notably nuclear factor erythroid 2-related factor 2 (NRF2) downregulation—and a "microbial dysbiosis" toward pathogenic microbial complexes. However, current data primarily identify mechanistic intersections rather than definitive causality, with the "PCOS-specific" impact often confounded by shared metabolic risk factors like insulin resistance and adiposity. Mapping this evidence reveals a profound overlap between PCOS and periodontitis through shared pathophysiological networks. Advancing this field requires a Core Outcome Set (COS) and well-designed randomized controlled trials. Clinically, recognizing periodontal disease as a potential contributor to the systemic inflammatory burden suggests that integrating routine.

**Supplementary Information:**

The online version contains supplementary material available at https://doi.org/10.1007/s44445-026-00254-9.

## Introduction

As a chronic inflammatory condition, periodontitis progressively damages the tissues that anchor the teeth—the gingiva, alveolar bone, and periodontal ligament. A bacterial biofilm initiates this process, but how quickly destruction proceeds depends heavily on host-related environmental and genetic factors. The consequences extend well past the mouth: periodontitis contributes to an individual’s overall systemic inflammatory load, a factor increasingly implicated in comorbidities such as atherosclerosis and diabetes mellitus (Kinane et al. 2017). Globally, the disease burden is substantial—surveillance data put the figure at roughly 42% of dentate U.S. adults aged 30 and above (Eke et al. 2018).

Crucially, the condition is no longer viewed as a localized oral issue (Hu et al. 2023). The inflamed periodontium serves as a reservoir for inflammatory mediators and pathogens that enter the systemic circulation (Damodaran et al. 2025; Helmi et al. 2015). This process is hypothesized to contribute to the overall systemic inflammatory burden, thereby suggesting a biological intersection between local periodontal destruction and systemic conditions such as PCOS. Ultimately, this proposes a plausible association between periodontal disease and broader systemic metabolic disorders (Porwal et al. 2014; Tewari et al. 2017; Zia et al. 2022).

Among reproductive-age women, PCOS ranks as one of the most common endocrine conditions, with global prevalence estimates around 10–13% (Teede et al. 2023). The disorder’s clinical presentation—hyperandrogenism, ovulatory dysfunction, and/or polycystic ovarian morphology (Smet and McLennan 2018)—typically co-occurs with metabolic disturbances such as insulin resistance, dyslipidemia, central obesity, and impaired glucose tolerance (Azziz et al. 2016). A growing body of evidence now frames PCOS less as a purely reproductive condition and more as a systemic inflammatory-metabolic disorder. Consistent with this view, affected women show elevated circulating inflammatory cytokines, oxidative stress markers, and endothelial dysfunction—signals that track with heightened long-term cardiovascular and metabolic risk (Azziz et al. 2016; González et al. 2012).

Periodontitis and PCOS share several underlying features—chronic low-grade inflammation, oxidative stress, and metabolic and microbial dysregulation—which has led researchers to propose a bidirectional relationship between the two. Pathways such as insulin resistance, exaggerated inflammatory responses, and dysregulated immunity may plausibly worsen disease severity in individuals who have both conditions (Cekici et al. 2014). That said, most supporting evidence to date is associative rather than causal, and the direction of these interactions has not been firmly established. Clarifying these mechanistic links matters for determining whether periodontal disease acts as a modifiable contributor to the systemic inflammatory profile seen in PCOS.

While individual studies have identified disparate biological links between PCOS and periodontal disease, these findings remain heavily fragmented across specialized disciplines, precluding a unified, systems-level understanding. Existing evidence is characterized by significant methodological heterogeneity, including differences in biomarkers assessed, biological compartments sampled, and analytical approaches. This lack of interdisciplinary synthesis obscures the potential bidirectional interactions between systemic endocrine dysregulation and local proteolytic destruction, making it difficult to determine whether these conditions interact through a unique mechanistic axis or merely overlap due to shared systemic risk factors.

Consequently, a scoping review, rather than a narrow systematic review, is required to comprehensively map this heterogeneous research landscape. This methodology is uniquely suited to this emerging field, as it allows for the synthesis of evidence across diverse study designs, identifies critical knowledge gaps that currently preclude a quantitative meta-analysis, and maps the intersections of four core biological domains: inflammatory pathways, oxidative stress signatures, hormonal–metabolic alterations, and oral microbial shifts. Ultimately, this review aims to establish a conceptual foundation for targeted, interdisciplinary clinical management.

## Materials and methods

Reporting for this scoping review followed the Preferred Reporting Items for Systematic Reviews and Meta-Analyses extension for Scoping Reviews (PRISMA-ScR) checklist (Tricco et al. 2018), while the review process itself was structured around the Joanna Briggs Institute (JBI) methodology for scoping reviews (Peters et al. 2020). The research was conducted at the European Univertity to data collection; the protocol was registered with the Open Science Framework (OSF): https://doi.org/10.17605/OSF.IO/WE3XU.

### Eligibility criteria

Study eligibility depended on a defined set of inclusion and exclusion criteria built around the Population, Concept, and Context (PCC) framework, chosen to keep the selection process reproducible and transparent. Eligible records were limited to original human research—case-control, cross-sectional, cohort, or trial designs—published from 2010 through February 2026. Each included study also had to compare women with confirmed PCOS against a non-PCOS control group and report quantitative biomarker data alongside quantifiable clinical periodontal parameters.

Following the PCC framework, the specific criteria were defined as follows:

- **Population:** Reproductive-age women with PCOS diagnosed via established criteria (Rotterdam ESHRE/ASRM-sponsored PCOS consensus workshop (2004), the National Institutes of Health (NIH) criteria (1992) (Zawadski and Dunaif 1992), or the Androgen Excess and PCOS Society (AE-PCOS) criteria (Azziz et al. 2006). or a clearly documented clinical/biochemical diagnosis, compared to a non-PCOS control group.
- **Concept:** Quantitative data on biological mechanisms linking PCOS and periodontal health across four core domains: inflammatory, oxidative, hormonal-metabolic, and microbial. To define the specific scope of the evidence mapped, eligible biological parameters included inflammatory mediators (e.g., Interleukin-6 [IL-6], Tumor Necrosis Factor-alpha [TNF-α], high-sensitivity C-reactive protein [hs-CRP], Matrix Metalloproteinase-8 [MMP-8]), oxidative stress signatures (e.g., malondialdehyde [MDA], 8-hydroxy-2′-deoxyguanosine [8-OHdG], total antioxidant status [TAS]), hormonal-metabolic parameters (e.g., testosterone, luteinizing hormone [LH], Homeostatic Model Assessment for Insulin Resistance [HOMA-IR], lipid profiles, and Vitamin D), and microbial shifts (e.g., *Porphyromonas gingivalis*, *Fusobacterium nucleatum*). Furthermore, quantitative biochemical markers of bone turnover (e.g., C-terminal telopeptide [CTX], bone alkaline phosphatase [ALP]) were eligible for inclusion not as independent mechanistic pathways, but as quantifiable biological endpoints of local periodontal destruction.
- **Context:** Eligible clinical periodontal outcomes were exhaustively restricted to: Probing Depth (PD), Clinical Attachment Level (CAL), Bleeding on Probing (BOP)/Gingival Bleeding Index (GBI), Gingival Index (GI)/Modified Gingival Index (mGI), Plaque Index (PI)/Full-Mouth Plaque Score (FMPS), Periodontal Inflamed Surface Area (PISA), and Periodontal Epithelial Surface Area (PESA). Regarding periodontal case definitions, eligible studies were required to report the exact diagnostic thresholds used to classify periodontal disease. While established consensus frameworks (specifically the 2017 World Workshop Classification, CDC/AAP definitions, or the 1999 International Workshop criteria) were preferred, studies utilizing explicitly reported, author-defined clinical thresholds were also eligible for inclusion. To capture all available mechanistic evidence, this scoping review deliberately embraced diagnostic heterogeneity, systematically addressing the variance within our narrative synthesis.

Regarding language requirements, our protocol restricted full texts to English but permitted screening of non-English abstracts. We formally deviated from this restriction to include one translated Chinese longitudinal study (Dangli et al. 2024) due to the severe lack of longitudinal evidence, and no other non-English records met our inclusion criteria. Crucially, to meet the criteria for ‘confirmed PCOS,’ studies were required to diagnose the condition using established consensus frameworks (e.g., Rotterdam 2003, NIH 1992, or AE-PCOS 2006) or clearly documented clinical/biochemical diagnosis; studies relying solely on unverified, self-reported patient histories were strictly excluded. Furthermore, to eliminate systemic inflammatory confounding, we excluded studies involving populations with periodontal-altering systemic comorbidities, explicitly defined as: impaired glucose tolerance, cardiovascular diseases, thyroid or adrenal dysfunction, autoimmune and inflammatory disorders, and malignancies. Studies were also excluded if they failed to control for major environmental or pharmacological modifiers of periodontitis, such as active pregnancy or the recent use of antibiotics, non-steroidal anti-inflammatory drugs (NSAIDs), or immunosuppressants.

The complete, explicit criteria used to establish the boundary between eligible and ineligible studies are detailed in Table 1.

**Table 1:** Explicit inclusion and exclusion criteria

Domain	Inclusion Criteria	Exclusion Criteria
Study Design	Original human research (case-control, cross-sectional, cohort, and randomized trials).	Animal or in vitro experiments, reviews, case reports, and abstracts.
Publication Date	Published between 2010 and February 2026.	Published before 2010 or after February 2026.
Population (PCOS)	Reproductive-age women with PCOS diagnosed via established criteria (e.g., Rotterdam 2004, NIH 1992, AE-PCOS 2006) or confirmed clinically/biochemically.	Studies lacking confirmed diagnostic criteria for PCOS. Participants with other periodontal-altering systemic co-morbidities.
Control Group	Presence of a non-PCOS control group (preferably age- and BMI-matched).	Studies lacking a healthy, non-PCOS control group.
Clinical Outcomes (Periodontal)	Reporting of at least one quantifiable periodontal clinical parameter (e.g., PD, CAL, GI, or staging).	Studies lacking clinical periodontal assessments.
Mechanistic Outcomes (Biomarkers)	Reporting of at least one quantitative biological biomarker measurement linking PCOS and periodontal health (across inflammatory, oxidative, hormonal-metabolic, or microbial domains).	Studies lacking quantitative biomarker data.
Language	English full-text availability. (Note: One Chinese-language study, Dangli et al. 2024, was included as a documented protocol deviation due to critical data scarcity)	Non-English full texts.

Abbreviations: AE-PCOS, Androgen Excess and Polycystic Ovary Syndrome Society; BMI, Body Mass Index; CAL, Clinical Attachment Level; GI, Gingival Index; NIH, National Institutes of Health; PCOS, Polycystic Ovary Syndrome; PD, Probing Depth; Rotterdam 2004, Rotterdam ESHRE/ASRM-Sponsored PCOS Consensus Workshop Group criteria

### Information sources and search strategy

The final search strategy was developed in consultation with a biomedical librarian. A comprehensive search was conducted across **PubMed, Scopus, EBSCO, and Google Scholar** by **M.N and Y.Z** on February 28, 2026. The search strategy was structured around three core concept blocks combined using the Boolean operator ‘AND’. Block 1 pertained to the population (PCOS), Block 2 covered the clinical condition (periodontal health), and Block 3 focused on the overarching biological mechanisms. Within each block, relevant controlled vocabulary (e.g., MeSH terms) and free-text terms in the title/abstract fields were combined using the Boolean operator ‘OR’. Specifically, Block 1 utilized terms such as ‘Polycystic Ovary Syndrome’[MeSH], ‘Polycystic Ovary Syndrome’, and ‘PCOS’. Block 2 included ‘Periodontal Diseases’[MeSH], ‘periodontitis’, ‘gingivitis’, ‘periodontal status’, and ‘oral health’. Block 3 utilized ‘Biomarkers’[MeSH], ‘Inflammation’[MeSH], ‘Oxidative Stress’[MeSH], and ‘Microbiota’[MeSH], alongside free-text terms including ‘biomarker*’, ‘cytokine*’, ‘oxidative’, ‘microbiom*’, and ‘dysbiosis’. Initially, a pilot search in PubMed was performed and refined to optimize sensitivity and precision. The full search string, which was adapted for other databases using platform-specific syntax, was peer-reviewed in accordance with the PRESS (Peer Review of Electronic Search Strategies) checklist principles (McGowan et al. 2016) and is available in its entirety in the Search string supplementary file.

To ensure comprehensive coverage of grey literature while maintaining search precision, Google Scholar screening was focused on the first 200 relevance-sorted records. This pragmatic threshold was informed by evidence synthesis standards suggesting that primary relevant data typically populate initial results. Yield saturation was continuously monitored during the process; no additional unique eligible studies were identified after the first 100 results, confirming that the 200-record cap was sufficient to capture all relevant literature within this engine. Additionally, the reference lists of both selected studies and related systematic reviews were screened for further relevant records to ensure comprehensive coverage.

### Study selection and screening

Two independent reviewers, **M.N and Y.Z** selected studies in two phases. Retrieved records were managed in Zotero, where duplicates were removed automatically and verified manually. In Phase 1, reviewers screened titles and abstracts; in Phase 2, they assessed retrieved full texts for final inclusion. Prior to screening, the reviewers piloted 20 records to ensure consistency, targeting ≥90% inter-rater agreement. Discrepancies were resolved through consensus or a third reviewer. All decisions were documented in a standardized spreadsheet to ensure independent assessment and transparent resolution.

### Data extraction and charting

A standardized Excel extraction form was piloted on three representative studies. Two independent reviewers, **M.N and Y.Z** extracted data, cross-checking a random 20% sample to ensure accuracy. Extracted variables included study characteristics (author, year, design), demographics (age, PCOS criteria, confounders), periodontal assessments (American Academy of Periodontology/Centers for Disease Control and Prevention [AAP/CDC] definitions, examiner calibration, outcomes like PD, CAL, BOP), and biomarker data (type, sample source [serum, saliva, GCF], direction, and statistical significance). To facilitate narrative synthesis, data were mapped into four thematic domains: (1) inflammatory pathways, (2) oxidative stress signatures, (3) hormonal–metabolic interactions, and (4) oral microbial shifts. This framework was used to identify shared pathophysiological networks and key mechanistic intersections—such as systemic inflammatory priming and oral dysbiosis—linking PCOS and periodontal disease.

### Quality assessment

To contextualize evidence quality, study-specific Critical Appraisal Skills Programme (CASP) checklists were applied (Critical Appraisal Skills Programme 2018): the Randomized Controlled Trial Checklist for the trial, the Cohort Study Checklist for prospective longitudinal designs, and the Case-Control Study Checklist for case-control and cross-sectional studies (selected to evaluate observational bias and confounding). Following JBI guidelines (Aromataris et al. 2024), appraisal-informed interpretation mapped the literature comprehensively without using quality as an exclusion criterion. Two independent reviewers evaluated each study across three domains (internal validity, results, and local applicability), resolving discrepancies through discussion. Scores weighted evidence strength during narrative synthesis to highlight methodological gaps.

### Use of artificial intelligence

This manuscript utilized specialized artificial intelligence applications, specifically NotebookLM (a large language model-based tool) for proofreading and language polishing of the human-generated text, and DeepL (a neural machine translation system) for translation assistance. No other generative AI was employed to create the manuscript content. All AI-assisted outputs were rigorously reviewed and validated by the authors, who assume full responsibility for the integrity of the final work.

## Results

### Selection of sources of evidence

The systematic search across the four databases and hand-searching of reference lists identified 86 records. After removal of 13 duplicates, 73 records underwent title and abstract screening. Of these, 33 records were excluded, primarily due to absence of periodontal assessment or dental outcomes (*n* = 13), lack of mechanistic biomarker data relevant to the review question (*n* = 5), incorrect study design or publication type (*n* = 7), ineligible population or comparator (*n* = 7), and unrelated topic (*n* = 1). The remaining 40 reports were sought for full-text retrieval.

**Fig. 1:**
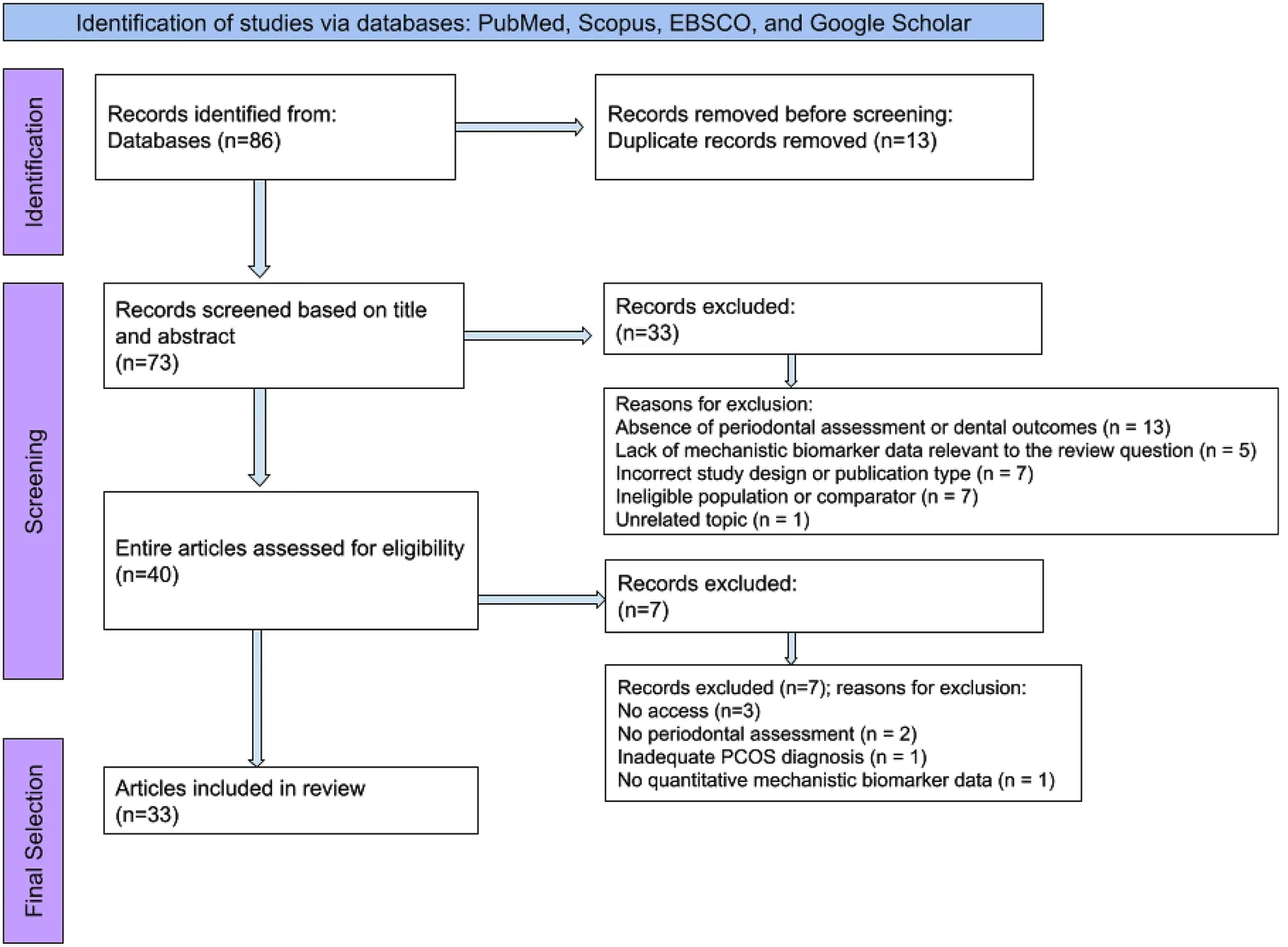
PRISMA flow diagram illustrating the systematic selection process for the scoping review. The flowchart details the identification of 86 records across four databases (PubMed, Scopus, EBSCO, and Google Scholar). Following the removal of 13 duplicate records, 73 records underwent title and abstract screening, resulting in 33 exclusions. Of the remaining 40 articles assessed for full-text eligibility, 7 were excluded (including 3 due to lack of access), resulting in a final total of 33 studies included in the evidence synthesis

At the full-text stage, three reports were excluded because the full texts could not be retrieved, leaving 37 full-text articles for eligibility assessment. Of these, four articles were excluded for the following reasons: absence of periodontal assessment (*n* = 2), inadequate PCOS diagnosis due to reliance on self-reported rather than established clinical diagnostic criteria (*n* = 1), and absence of quantitative mechanistic biomarker data (*n* = 1) (Supplementary Table 1). Ultimately, 33 studies met all inclusion criteria and were included in the evidence synthesis (Achu Joseph et al. 2023; Akcalı et al. 2014, 2015, 2017; Damodaran et al. 2025; Dangli et al. 2024; Dharuman et al. 2022; Dursun et al. 2011; Hameed and Ahmed 2017; Helmi et al. 2015; Hu et al. 2023; Işık et al. 2020; Liu et al. 2024; Maboudi et al. 2023; Márquez-Arrico et al. 2025a, b; Najafi et al. 2017; Özçaka et al. 2012, 2013; Porwal et al. 2014; Rahiminejad et al. 2015; Saglam et al. 2018, 2020; Sahithi et al. 2025; Saljoughi et al. 2020; Tahir and Hussien 2017, 2019; Tewari et al. 2017; Varadan et al. 2019; Wendland et al. 2021a, b; Yasar et al. 2025; Zia et al. 2022). Study selection is presented in the PRISMA flow diagram (Fig. 1).

### Characteristics of sources of evidence

Thirty-three studies were included in the evidence synthesis. Study characteristics are summarized in (Supplementary Table 2). The majority of studies employed case-control (*n* = 17 (Achu Joseph et al. 2023; Akcalı et al. 2014, 2015; Hameed and Ahmed 2017; Helmi et al. 2015; Hu et al. 2023; Liu et al. 2024; Maboudi et al. 2023; Márquez-Arrico et al. 2025a, b; Özçaka et al. 2013; Saljoughi et al. 2020; Tahir and Hussien 2017, 2019; Varadan et al. 2019; Wendland et al. 2021a; Zia et al. 2022); or cross-sectional (*n* = 10 (Akcalı et al. 2017; Damodaran et al. 2025; Dharuman et al. 2022; Dursun et al. 2011; Najafi et al. 2017; Porwal et al. 2014; Rahiminejad et al. 2015; Saglam et al. 2018, 2020; Sahithi et al. 2025).; Notably, three additional studies utilized a case-control recruitment framework but were cross-sectional in their assessment of outcomes (*n* = 3; Işık et al. 2020; Özçaka et al. 2012; Wendland et al. 2021b), as they utilized purposive recruitment of distinct disease and healthy cohorts to measure biomarkers at a single point in time. This reinforces the predominance of observational, non-longitudinal evidence (totaling 30 of 33 studies) within the current literature. Only one randomized controlled trial (Tewari et al. 2017) and two prospective longitudinal investigations (Dangli et al. 2024; Yasar et al. 2025) were identified.

Sample sizes ranged from 40 to 206 participants. Geographically, the included studies were predominantly conducted in Asia and the Middle East, including India *n* = 8 (Achu Joseph et al. 2023; Damodaran et al. 2025; Dharuman et al. 2022; Porwal et al. 2014; Sahithi et al. 2025; Tewari et al. 2017; Varadan et al. 2019; Zia et al. 2022), Turkey *n* = 8 (Akcalı et al. 2014, 2015, 2017; Dursun et al. 2011; Işık et al. 2020; Saglam et al. 2018, 2020; Yasar et al. 2025), Iran *n* = 4 (Maboudi et al. 2023; Najafi et al. 2017; Rahiminejad et al. 2015; Saljoughi et al. 2020), Iraq *n* = 4 (Hameed and Ahmed 2017; Helmi et al. 2015; Tahir and Hussien 2017, 2019), China *n* = 3 (Dangli et al. 2024; Hu et al. 2023; Liu et al. 2024), with additional contributions from Europe, specifically Poland (*n* = 2; Wendland et al. 2021a, b), and Spain (*n* = 2; Márquez-Arrico et al. 2025a, b). Two studies reported dual institutional affiliation, specifically Turkey and the United Kingdom (Özçaka et al. 2012, 2013).

Across studies, the Rotterdam criteria were most commonly used to establish PCOS diagnosis (*n* = 32; The Rotterdam ESHRE/ASRM-sponsored PCOS consensus workshop group 2004), with one study applying the AE-PCOS criteria (Azziz et al. 2006). Periodontal status was assessed using standardized clinical parameters, including PD, CAL, GI, bleeding on probing (BOP), and plaque index (PI). Methodologically, nine studies applied the 2017 World Workshop classification (Papapanou et al. 2018), seven used CDC/AAP case definitions (Eke et al. 2012), and six employed earlier classification frameworks such as the 1999 International Workshop or AAP 1998–1999 criteria (Armitage 1999). Eleven studies reported clinical parameters without explicitly citing a formal classification system or used author-defined thresholds for periodontal case definitions. To manage this methodological heterogeneity during synthesis, we adopted a narrative mapping approach, categorizing biological findings into four thematic domains. This focused the synthesis on consistent mechanistic patterns across studies rather than disparate clinical thresholds. Furthermore, data interpretation was contextualized using CASP quality appraisals to weight the evidence, and this identified diversity was utilized to justify the need for a future standardized Core Outcome Set (COS). Most studies controlled for Body Mass Index (BMI) to isolate the independent impact of PCOS on periodontal health. Specifically, 16 studies (e.g., Akcalı et al. 2014; Yasar et al. 2025) utilized BMI-matching between cohorts to ensure comparability. Furthermore, 19 studies managed this confounder by excluding participants outside predefined ranges—most commonly those with obesity (typically BMI > 30 kg/m^2^) or underweight status (e.g., Hu et al. 2023; Wendland et al. 2021a).

Quality appraisal conducted via CASP checklists demonstrated generally high methodological quality across the included studies, with most criteria receiving ‘Yes’ responses. Lower scores were primarily associated with cross-sectional designs, particularly regarding power calculations and the reporting of statistical significance.

### Evidence distribution across mechanistic domains

The extracted data were mapped across the four mechanistic domains defined in the protocol: (1) inflammatory markers, (2) oxidative stress signatures, (3) hormonal–metabolic pathways, and (4) oral microbial shifts. It should be noted that several studies have performed interdisciplinary assessments across multiple biological pathways. The principal biomarkers across the mechanistic domains have been summarized in (Supplementary Table 3).

Inflammatory biomarkers were assessed in 17 of 33 studies (51.5% (Akcalı et al. 2015, 2017; Dangli et al. 2024; Helmi et al. 2015; Hu et al. 2023; Maboudi et al. 2023; Márquez-Arrico et al. 2025a, b; Özçaka et al. 2012, 2013; Porwal et al. 2014; Saglam et al. 2020; Saljoughi et al. 2020; Tahir and Hussien 2019; Tewari et al. 2017; Wendland et al. 2021a; Yasar et al. 2025). Hormonal–metabolic parameters were investigated in 15 studies (45.5% (Dangli et al. 2024; Helmi et al. 2015; Hu et al. 2023; Işık et al. 2020; Liu et al. 2024; Márquez-Arrico et al. 2025a; Özçaka et al. 2013; Rahiminejad et al. 2015; Sahithi et al. 2025; Saljoughi et al. 2020; Tahir and Hussien 2017; Tewari et al. 2017; Varadan et al. 2019; Wendland et al. 2021a, b); Oxidative stress markers were evaluated in 10 studies (30.3%; Damodaran et al. 2025; Dharuman et al. 2022; Dursun et al. 2011; Hameed and Ahmed 2017; Márquez-Arrico et al. 2025a, b; Najafi et al. 2017; Rahiminejad et al. 2015; Saglam et al. 2018; Varadan et al. 2019). Microbial investigations were the least represented domain, identified in only 3 of 33 studies (9.1%; Achu Joseph et al. 2023; Akcalı et al. 2014; Wendland et al. 2021b). Due to this limited representation, the current evidence regarding the oral microbiome’s role in the PCOS–periodontal axis remains preliminary. One study (Zia et al. 2022) focused on bone metabolism markers (e.g., CTX, bone alkaline phosphatase) rather than the four primary domains. To ensure a cohesive synthesis, this data was integrated as a downstream clinical outcome of the hormonal–metabolic and inflammatory axes. Bone metabolism is not treated as a standalone fifth pathway in this review but is presented as the structural endpoint where systemic endocrine dysregulation translates into localized alveolar bone loss. The biological biomarkers investigated and corresponding periodontal clinical findings across all included studies are detailed in (Supplementary Table 4).

## Discussion

### Interpretation of evidence patterns across mechanistic domains

When interpreting these mapped networks, it is critical to distinguish evidence certainty. Inflammatory and oxidative stress domains (representing 17 and 10 studies, respectively) demonstrate highly consistent associative baselines. Conversely, hormonal-metabolic data exhibit greater variability due to unstandardized sampling, and the microbial dimension remains highly preliminary, supported by only three studies. Across all domains, evidence supporting definitive, bidirectional causality remains strictly limited.

#### Inflammatory pathway convergence

The mapped evidence of the inflammatory pathways connecting PCOS and periodontal disease primarily reveals observational associations rather than definitive causal mechanisms. Studies consistently report a systemic baseline of elevated pro-inflammatory mediators—such as IL-6, TNF-α, and Matrix Metalloproteinase-8 (MMP-8)—in women with PCOS (Akcalı et al. 2015; Özçaka et al. 2012). This systemic burden is further characterized by chronic endothelial inflammation, evidenced by elevated serum Endocan (Saglam et al. 2020) and hyper-activated leukocyte–endothelium interactions (Márquez-Arrico et al. 2025a). While these patterns suggest a systemic low-grade inflammatory state that lowers the threshold for local tissue destruction, the predominance of cross-sectional data requires a cautious interpretation regarding temporality. Rather than viewing these as definitive causal links, the identified intracellular ‘priming and assembly’ models mediated by the NLRP3 inflammasome, NF-κB, and JNK should be considered proposed mechanistic intersections; linking these molecular pathways to clinical inflammatory markers like bleeding on probing (BOP) represents an extrapolation of largely correlative data (Márquez-Arrico et al. 2025).

A key limitation in the existing literature is the tendency to overinterpret localized biomarker fluctuations as proof of a self-perpetuating, bidirectional disease loop. While rare longitudinal data suggest that a surge in salivary IL-6 and MMP-8 mirrors the worsening of PD and CAL (Dangli et al. 2024), and markers like Interleukin 17-A (IL-17A) and Interleukin 17-F (IL-17F) correlate positively with both periodontal destruction and hirsutism scores (Özçaka et al. 2013), current observational data lack the mechanistic validation needed to distinguish whether this is a true PCOS-specific pathway or merely a reflection of shared metabolic risk factors like insulin resistance. Highlighting the need to explore this nuance, recent evidence shows divergent responses in local pro-resolving mediators—such as a unique increase in gingival Annexin A1 (ANXA1) observed in PCOS patients following periodontal therapy—suggesting that subtle, hormone-modulated immune dynamics specific to the syndrome may still exist (Yasar et al. 2025).

#### Oxidative stress as a shared mechanism

Included studies reveal a consistent oxidative stress pattern in women with PCOS, independent of periodontal status (Saglam et al. 2018). Observational data show elevated systemic and local pro-oxidants—including malondialdehyde (MDA), 8-hydroxy-2′-deoxyguanosine (8-OHdG), Advanced Oxidation Protein Products (AOPP), and Advanced Glycation End products (AGEs) (Damodaran et al. 2025; Dharuman et al. 2022; Varadan et al. 2019). Concurrently, antioxidant defenses appear compromised, evidenced by reduced glutathione, diminished total antioxidant status (TAS) (Márquez-Arrico et al. 2025a), and downregulation of the NRF2–GCLC–GSR pathway (Márquez-Arrico et al. 2025b). When PCOS and periodontitis coexist, comorbid groups exhibit amplified oxidative signatures with peak levels of AGEs, homocysteine, and nitric oxide (NO) (Damodaran et al. 2025; Hameed and Ahmed 2017), alongside salivary myeloperoxidase (MPO) correlating strongly with PD and BOP (Akcalı et al. 2017).

However, caution is required when extrapolating static biomarker elevations into causal loops. The hypothesis that reactive oxygen species (ROS) trigger NLRP3 inflammasome assembly to drive tissue breakdown remains theoretical due to the cross-sectional nature of current evidence (Márquez-Arrico et al. 2025b). A critical gap is distinguishing whether these markers represent a unique, PCOS-driven pathway of localized destruction or merely reflect shared systemic adiposity or insulin resistance. Additionally, local and systemic oxidative states do not always align; for example, NO levels can be elevated in GCF but normal in serum (Dursun et al. 2011). Finally, there is a distinct lack of data on receptor-level drivers, specifically the RAGE signaling axis, which likely mediates how these accumulated oxidative products translate into clinical tissue loss.

#### Hormonal–metabolic interactions

Hormonal shifts and metabolic dysregulation in PCOS play key roles in periodontal progression. Observational studies show that systemic androgen elevations (total and free testosterone) and altered gonadotropin dynamics (LH, LH/FSH ratio) correlate positively with periodontal destruction (PD, CAL) and proteolytic activity (MMP-8) (Dangli et al. 2024; Hu et al. 2023). Simultaneously, metabolic parameters—specifically HOMA-IR and dyslipidemia (low-density lipoprotein cholesterol [LDL-C] and triglycerides)—parallel subgingival inflammation severity (Sahithi et al. 2025; Tewari et al. 2017).

However, these associations are often oversimplified as static snapshots. The hypothesis that hyperandrogenism directly “primes” the periodontium ignores potential biphasic effects; indeed, a negative correlation between salivary testosterone and inflammation in adolescents suggests a poorly understood biological window of androgen signaling (Wendland et al. 2021a). Furthermore, assuming serum concentrations reflect local tissue utilization remains a major limitation.

A significant mechanistic gap is the total absence of highly specific biomarkers—such as Anti-Müllerian Hormone (AMH) (Dewailly et al. 2014) and Adiponectin—within the 33 included studies. Without measuring AMH, the primary marker of follicular arrest, it is unclear if oral destruction scales with reproductive severity. Similarly, the role of Adiponectin, a protective anti-inflammatory adipokine, remains unexplored, leaving a void in our understanding of what drives the exaggerated bone resorption—evidenced by altered bone mineral density and turnover markers like C-terminal telopeptide (CTX) and bone alkaline phosphatase—observed in PCOS cohorts (Zia et al. 2022).

#### Microbial dimension

Evidence mapping of the microbial dimension remains limited; only three included studies examined the oral microbiome (Achu Joseph et al. 2023; Akcalı et al. 2014; Wendland et al. 2021b). Nonetheless, available data suggest subgingival pathogen colonization and elevated antibody responses to *Porphyromonas gingivalis*, *Tannerella forsythia*, and *Fusobacterium nucleatum* in PCOS cohorts (Achu Joseph et al. 2023; Akcalı et al. 2014).

Biologically, hyperandrogenism and altered steroids may modulate this shift. Specifically, progesterone and estrogen can substitute for Vitamin K as essential growth factors for black-pigmented anaerobes like *Prevotella intermedia* (Kornman and Loesche 1982). These dysbiotic shifts also correlate with metabolic status: subgingival *Capnocytophaga gingivalis* correlates positively with systemic estradiol, while *Aggregatibacter actinomycetemcomitans* and orange complex bacteria correlate negatively with HDL-C (Wendland et al. 2021b).

While valuable, we must evaluate whether these microbial shifts are directly driven by PCOS-specific hormonal derangements. While *F. nucleatum* acts as a biofilm bridge (Socransky et al. 1998) and *P. gingivalis* gingipains activate TLR-4 pathways (Watanabe et al. 2011), direct links to a unique PCOS mechanism remain unproven. Dysbiosis may simply be a secondary byproduct of systemic inflammation and altered salivary flow. Highlighting this, adolescent PCOS cohorts (aged 15–19) do not exhibit elevated subgingival pathogenicity compared to controls (Wendland et al. 2021b). This age-dependent divergence suggests that early-life endocrine and metabolic pathways are not yet mature enough to drive pathogenic complexes, which likely develop only after prolonged adult exposure.

Finally, a critical gap remains the lack of unbiased ecological mapping. Relying on targeted PCR panels prevents the identification of syndrome-specific signatures that 16S rRNA or shotgun metagenomics could reveal. Furthermore, the hypothesized “Oral–Gut–Systemic Axis”—where translocated oral pathobionts may contribute to insulin resistance—remains entirely unvalidated due to a lack of paired oral and stool sampling (Li et al. 2021, 2025).

### Methodological heterogeneity and standardization needs

A primary challenge in the current literature is profound methodological heterogeneity. Although 97% of the included studies utilized the 2003 Rotterdam criteria, the widespread failure to report specific PCOS phenotypes prevents isolating whether periodontal destruction is driven by androgen excess, metabolic dysfunction, or their synergy—even though recent evidence demonstrating varied gingival bleeding scores across distinct subtypes highlights the critical need for this stratification (Liu et al. 2024). Similarly, reliance on disparate periodontal diagnostic frameworks profoundly complicates the direct comparison of disease severity across the literature. The included studies span the 1999 criteria, CDC/AAP definitions, and the 2017 World Workshop Classification. Because these systems operate on fundamentally different diagnostic algorithms—ranging from strict linear probing depth cut-offs to multidimensional staging and grading—establishing a standardized disease threshold is precluded. Compounding this issue, one-third of the included studies (*n* = 11) relied entirely on unstandardized, author-defined thresholds, preventing the identification of a universal clinical baseline for exactly when localized inflammation begins to significantly exacerbate the systemic burden in PCOS. Furthermore, unstandardized biochemical sampling across serum, saliva, and GCF introduces “biological noise,” as unpaired markers cannot adequately distinguish the systemic inflammatory load from localized tissue destruction.

Methodological limitations identified during our CASP appraisal—specifically the overwhelming predominance of cross-sectional designs, a pervasive lack of a priori power calculations, inconsistent adjustment for metabolic confounders like adiposity and insulin resistance—alongside a widespread failure to distinguish between lean and obese women—significantly dilute the strength of proposed causal loops. Consequently, the current literature restricts our confidence in bidirectional causality, meaning these findings can only reliably support associative mapping rather than definitive, directional mechanisms. To transition from observational mapping to actionable clinical guidelines, future research must establish a Core Outcome Set (**COS)**. This standardized framework should mandate the 2017 World Workshop periodontal classification, explicit PCOS phenotypic stratification, and paired local-systemic biological sampling (e.g., simultaneous GCF and serum analysis).

### Interventional frontiers and pharmacological management of reproductive outcomes

The overarching limitation in PCOS–periodontitis research is the profound scarcity of longitudinal and interventional data. The predominance of cross-sectional studies restricts confirming bidirectional causality, necessitating a shift toward randomized controlled trials (RCTs) evaluating combined systemic and localized therapies. Preliminary data highlight this synergy: combining non-surgical periodontal therapy with myo-inositol yields significantly greater reductions in systemic hs-CRP than medical therapy alone (Tewari et al. 2017). Furthermore, patients receiving established PCOS medical treatments (e.g., insulin sensitizers, oral contraceptives) exhibit significantly less periodontal breakdown and systemic inflammation than untreated cohorts, suggesting pharmacological management stabilizes the systemic inflammatory environment (Porwal et al. 2014).

To bridge existing gaps, future RCTs must prioritize high-impact reproductive endpoints, evaluating whether periodontal therapy directly enhances ovulation and conception rates. Validating these outcomes requires transitioning from narrow assays to multi-omic approaches and high-throughput, non-invasive profiling—specifically Raman spectroscopy (Hu et al. 2023)—to monitor real-time molecular responses. Integrating these “deep-phenotyping” tools will provide the definitive evidence to evaluate whether routine periodontal screening should be incorporated into interdisciplinary PCOS management.

### Clinical and interdisciplinary implications

The PCOS-periodontal overlap supports the biological plausibility of a highly integrated care model as a future clinical consideration. Early screening may be particularly beneficial during adolescence, when pro-inflammatory cytokines are already detectable, to potentially manage disease progression (Opydo-Szymaczek et al. 2025). Clinically, endocrinologists might consider incorporating periodontal assessments into routine evaluations, while dentists could view unexplained gingival inflammation in young, non-obese females as a potential indicator of endocrine dysfunction warranting medical referral (Hu et al. 2023). Furthermore, interventions like professional mechanical plaque removal (PMPR) can effectively reduce salivary IL-6, indicating that local oral healing is achievable despite underlying hormonal imbalances (Yasar et al. 2025). Addressing this modifiable inflammatory target may help mitigate long-term cardiometabolic risks (Márquez-Arrico et al. 2025a), underscoring the utility of coordinated interdisciplinary management to optimize metabolic and reproductive health.

### Strengths and limitations

Several strengths distinguish this review. Findings from the inflammatory, oxidative, hormonal-metabolic, and microbial domains are drawn together into a systems-level, bidirectional view of the PCOS–periodontal axis. Because the synthesis spans periodontology, endocrinology, and immunology, it points toward translational implications for integrated patient care that a single-discipline review would likely miss. Nevertheless, several limitations must be considered:

- **Study Designs:** The evidence base relies heavily on static data; 27 of the 33 included studies utilize cross-sectional or case-control designs. A profound scarcity of longitudinal data (only two prospective studies and one trial) prevents establishing causality or temporal direction.
- **Generalizability:** Many studies were single-center, utilized small samples (40–206 participants), and originated from a limited geographic range (predominantly Asia and the Middle East).
- **Methodological Heterogeneity:** Direct comparisons are complicated because one-third of the studies failed to use a formal periodontal classification, and laboratory assay and biomarker quantification techniques varied widely.
- **Confounding Baselines:** Highly underexplored domains persist, particularly the microbiological axis (only three studies). Because both disorders share chronic inflammation, insulin resistance, and oxidative stress, current associative data cannot definitively separate a unique, PCOS-specific pathway from the cumulative burden of shared systemic risk factors.
- **Search and Language Constraints:** Searching only four databases and primarily English publications limits exhaustive mapping. Excluding a few papers due to a lack of full-text access may affect completeness. Translating one highly relevant non-English longitudinal study represents a documented deviation from our English-only full-text criterion.
- **Publication Bias:** A tendency toward publishing statistically significant positive findings may artificially overrepresent the strength of this bidirectional axis.

The goal of this scoping review was to provide a qualitative map of the existing literature rather than a statistical meta-analysis. Consequently, this study does not provide pooled effect sizes, but establishes the necessary framework and identifies critical evidence gaps to guide future systematic investigations.

## Conclusion

Current evidence mapping reveals a profound, multidimensional overlap between PCOS and periodontal disease. Rather than a simple linear relationship, the mapped literature suggests a theoretical framework where the two conditions may contribute to one another through a shared network of immunoendocrine dysfunction, oxidative stress, and metabolic dysregulation. However, the existing literature is heavily dominated by cross-sectional observations, meaning that the frequently proposed bidirectional, causal loops remain largely theoretical.

To advance this field, future research must shift from repetitive associative mapping toward standardized, interventional models. Establishing a COS, utilizing paired local-systemic biological sampling, and investigating distinct pharmacological and reproductive endpoints will clarify whether periodontal destruction is driven by a unique PCOS-specific mechanism or represents the cumulative burden of shared systemic risk factors.

Clinically, the recognition of periodontal disease as a highly modifiable source of chronic inflammation suggests a promising, yet-to-be-validated avenue for PCOS management. Preliminary evidence suggests that controlling local oral biofilms may dampen the systemic inflammatory burden of the syndrome; however, given that the current evidence base is predominantly cross-sectional, these findings must be viewed as hypothesis-generating rather than definitive. Ultimately, bridging these knowledge gaps requires a transition toward rigorous interventional models to determine if evaluating periodontal screening as a future clinical consideration within the interdisciplinary management of women with PCOS can effectively optimize long-term cardiometabolic and reproductive outcomes.

## Electronic supplementary material

Below is the link to the electronic supplementary material.

Supplementary Material 1

Supplementary Material 2

## Data Availability

All data generated or analyzed during this study are included in this published article, and the Supplementary Tables file. The primary data sources are the 33 original research studies identified through the systematic search, with extracted results synthesized and presented in the provided tables and figures.
