# Supplementary material for: Mechanistic insights into polycystic ovary syndrome and periodontal disease: a systematic scoping review of inflammatory, oxidative stress, hormonal–metabolic, and microbial pathways
Source: Saudi Dent J. 2026 Oct 26;38(11):141. doi: 10.1007/s44445-026-00254-9 (PMC13710495; doi:10.1007/s44445-026-00254-9)
Supplement: Supplementary file 1 [file 44445_2026_254_MOESM1_ESM.docx]

**PubMed**

("Polycystic Ovary Syndrome"[MeSH] OR "polycystic ovary syndrome"[tiab] OR PCOS[tiab])

AND

("Periodontal Diseases"[MeSH] OR periodontitis[tiab] OR gingivitis[tiab] OR "periodontal status"[tiab] OR "oral health"[tiab])

AND

("Biomarkers"[MeSH] OR "Inflammation"[MeSH] OR "Oxidative Stress"[MeSH] OR "Microbiota"[MeSH] OR biomarker[tiab] OR cytokine[tiab] OR oxidative[tiab] OR microbiom*[tiab] OR dysbiosis[tiab])

**Scopus**

TITLE-ABS-KEY(

"polycystic ovary syndrome"

OR PCOS

)

AND

TITLE-ABS-KEY(

periodontitis

OR gingivitis

OR "periodontal disease*"

OR "periodontal status"

OR "oral health"

)

AND

TITLE-ABS-KEY(

biomarker*

OR cytokine*

OR inflammation

OR "oxidative stress"

OR microbiome

OR microbiota

OR dysbiosis

OR "tumor necrosis factor"

OR interleukin*

)

**EBSCO**

(MH "Polycystic Ovary Syndrome" OR TI "polycystic ovary syndrome" OR AB "polycystic ovary syndrome" OR TI PCOS OR AB PCOS)

AND

(MH "Periodontal Diseases" OR TI periodontitis OR AB periodontitis OR TI gingivitis OR AB gingivitis OR TI "periodontal disease*" OR AB "periodontal disease*" OR TI "periodontal status" OR AB "periodontal status" OR TI "oral health" OR AB "oral health")

AND

(MH "Biomarkers" OR MH "Inflammation" OR MH "Oxidative Stress" OR MH "Microbiota" OR TI biomarker* OR AB biomarker* OR TI cytokine* OR AB cytokine* OR TI inflammation OR AB inflammation OR TI oxidative OR AB oxidative OR TI microbiom* OR AB microbiom* OR TI dysbiosis OR AB dysbiosis)

**Google Scholar**

"polycystic ovary syndrome" OR PCOS periodontitis OR gingivitis OR "periodontal disease" biomarkers OR cytokines OR inflammation OR "oxidative stress" OR microbiome
