# Supplementary material for: Mechanistic insights into polycystic ovary syndrome and periodontal disease: a systematic scoping review of inflammatory, oxidative stress, hormonal–metabolic, and microbial pathways
Source: Saudi Dent J. 2026 Oct 26;38(11):141. doi: 10.1007/s44445-026-00254-9 (PMC13710495; doi:10.1007/s44445-026-00254-9)
Supplement: Supplementary file 2 [file 44445_2026_254_MOESM2_ESM.docx]

**Tables**

**Supplementary Table 1: Reasons for exclusion at title/abstract and full-text screening**

**Supplementary Table 1.A. Studies excluded during title and abstract screening (n = 33) and their primary reasons for exclusion.** Each excluded record was assigned a single primary reason for exclusion in accordance with the study selection process.

| **Exclusion Category** | **n** |
| --- | --- |
| Incorrect study design/publication type  (Review articles, case report, unpublished clinical trial, or no results) | 7 |
| Absence of periodontal assessment/dental outcome  (No periodontal parameters, no dental association, or no clinical periodontal outcomes) | 13 |
| No mechanistic biomarker data relevant to review question  (Biomarkers absent, not the biomarkers of interest, or not linked to periodontal outcomes) | 5 |
| Population or comparator not eligible  (No PCOS population or no healthy control group) | 7 |
| Unrelated to review question | 1 |
| **Total** | **33** |

**Supplementary Table 1.B. Studies excluded following full-text eligibility assessment (n = 7) and the corresponding reasons for exclusion.** Each study was assigned one primary exclusion reason based on the predefined eligibility criteria.

| **Exclusion Category** | **n** |
| --- | --- |
| Full text unavailable | 3 |
| No periodontal assessment | 2 |
| No quantitative mechanistic biomarker data | 1 |
| Inadequate PCOS diagnosis (self-reported) | 1 |
| **Total** | **7** |

**Supplementary Table 2. Characteristics of the studies included in the scoping review.** The table summarizes study design, population characteristics, PCOS diagnostic criteria, periodontal assessment methods, and key methodological features. Abbreviations are defined below.

| **Title** | **Author, Year** | **Country** | **Study Design** | **Sample Size *(N)*** | **Age (Mean ± SD/ Range** | **Groups Compared** | **PCOS Diagnostic Criteria** | **Periodontal Diagnostic Criteria** | **Exclusion Criteria** |
| --- | --- | --- | --- | --- | --- | --- | --- | --- | --- |
| Annexin A1 levels in polycystic ovary syndrome: impact of professional mechanical plaque removal. | Yasar et al., 2025 | Turkey | Prospective clinical trial | 80 | 18–40 years (Median: 21–22.5) | PCOSH, PCOSG, SG, and SH | Rotterdam | 2017 WWC; Silness & Löe Index | Systemic disease; BMI>30; pregnancy; smoking; medications affecting periodontal/systemic health; recent periodontal or antimicrobial therapy; <20 teeth |
| Assessment of oral health status, leptin, and inflammatory markers in serum and saliva of patients with polycystic ovarian syndrome in reference to metabolic syndrome | Helmi et al., 2015 | Iraq | Case-control | 72 | PCOS: 27.9 ± 5.6, Control: 27.6 ± 5.8 | PCOS (n=42) vs. Healthy (n=30) | Rotterdam | GI, PI | Smoking; pregnancy; thyroid/adrenal dysfunction; DM; hypertension. |
| Association between metabolic and hormonal profile, proinflammatory cytokines in saliva and gingival health in adolescent females with polycystic ovary syndrome | Wendland et al., 2021a | Poland | Case-control | 59 | 15–19 years (Mean: ~16.3) | PCOS (n=31) vs. Healthy (n=28) | Rotterdam | 2017 WWC; GI, PI | Obesity; smoking; systemic/thyroid disorders; hormonal therapy; recent ATB; orthodontic appliances. |
| Association between Polycystic Ovary Syndrome, Oral Microbiota and Systemic Antibody Responses | Akcalı et al. 2014 | Turkey | Case-control | 125 | 18–40 (Mean: 25.9) | PCOSH (n=45); PCOSG (n=35); SH (n=25); SG (n=20) | Rotterdam | 1999 Workshop; GI (Löe, 1964); BOP >50% | BMI > 30 kg/m²; systemic diseases (DM, HTN, CVD); ATB, NSAIDs, or OCPs in the last 6 months |
| Bone markers and bone mineral density associates with periodontitis in females with poly‐cystic ovarian syndrome | Zia et al. 2022 | India | Case-control | 140 | 18–30 years (CP Group: 36.1 ± 10.3) | PCOS+CP (n=40); PCOS (n=40); CP (n=40); SH (n=20) | Rotterdam | 2017 WWC (AAP/EFP) | Thyroid/Adrenal dysfunction; tumours; smoking; OCPs, NSAIDs, or ATB in the last 6 months |
| Comparative analysis of serum and saliva samples using Raman spectroscopy: a high‐throughput investigation in patients with polycystic ovary syndrome and periodontitis | Hu et al., 2023 | China | Case-control | 88 | 20–34 years (Mean: ~25.8) | PCOS (n=66) vs.  Non-PCOS (n=22) | Rotterdam | 2017 WWC | Systemic disease; ATB (3 mos); periodontal therapy (12 mos); steroids, immunosuppressants, or bisphosphonates; ART |
| Comparison of prevalence of periodontal disease in women  with polycystic ovary syndrome and healthy controls | Rahiminejad et al., 2015 | Iran | Cross-sectional | 196 | 18–45 years (Mean: ~28.8) | PCOS (98) vs. Healthy (98) | Rotterdam | AAP (1999) | BMI > 25kg/m²; IGT; malignancy; osteoporosis; smoking; alcohol; periodontal therapy (6 mos) |
| Effect of Non-surgical Periodontal Therapy Along With Myo-Inositol on High Sensitivity C-Reactive Protein and Insulin Resistance in Polycystic Ovary Syndrome Women Having Chronic Periodontitis: A Randomized Controlled Trial | Deepti et al., 2017 | India | Randomized Controlled Trial | 85 | 15–35 years (Mean: ~23.3) | PCOS+CP (SRP+Inositol); PCOS+CP (Inositol only); SH | AES (2006) | Page & Eke (2007) | DM; CVD; renal failure; smoking; alcohol; OCPs or ATB (3 mos); recent periodontal/periapical treatment |
| Elevated matrix metalloproteinase-8 in saliva and serum in polycystic ovary syndrome and association with gingival inflammation | Akcalı et al. 2015 | Turkey | Case-control | 125 | 18–40 (Mean: 25.9) | PCOSH (n=45); PCOSG (n=35); SH (n=25); SG (n=20) | Rotterdam | 1999 Workshop; GI (Löe, 1964); BOP >50% | BMI >30kg/m²; systemic diseases (DM, HTN, CVD, Thyroid); OCPs, ATB, NSAIDs, or Insulin-sensitizers (6 mos) |
| Endocan and Interleukin-6 Levels in Individuals with polycystic ovary syndrome and periodontal inflammation: A prospective cross-sectional study | Saglam et al., 2020 | Turkey | Cross-sectional | 87 | 18–40 (Mean: ~26.0) | (1) PCOS+P (n=23); (2) PCOSH (n=23); (3) SHP (n=21); (4) SHPH (n=20) | Rotterdam | Armitage (1999); Page & Eke (2007) | BMI <18 or >25 kg/m²; HbA1c ≥ 6.5%; smoking; alcohol; periodontal therapy (6 mos); OCPs, ATB, or NSAIDs (3 mos) |
| Estimation of Salivary Advanced Glycation End Products in Polycystic Ovarian Syndrome Patients with and without Chronic Periodontitis: A Cross-sectional Study | Damodaran et al. 2025 | India | Cross-sectional | 48 | 18–35 years (Mean: ~26.5) | (1) PCOS+P (n=12); (2) PCOSH (n=12); (3) SHP (n=12); (4) SHPH (n=12) | Rotterdam | Page & Eke (2012) | Systemic disease; smoking; tobacco chewing; immunocompromised; ATB, NSAIDs, or periodontal therapy (6 mos) |
| Evaluation of Levels of Advanced Oxidative Protein Products in Patients with Polycystic Ovary Syndrome with and without Chronic Periodontitis: A Cross-Sectional Study | Dharuman et al., 2022 | India | Cross-sectional | 48 | 18–35 (Mean: 24.0) | (1) PCOS+CP (n=12); (2) PCOS (n=12); (3) CP (n=12); (4) SH (n=12) | Rotterdam | Page & Eke (2012) | Systemic disease; pregnancy; previous periodontal therapy; any medication use (3 mos); smoking |
| Evaluation of Microbial Profile in Patients with Polycystic Ovary Syndrome and Periodontal Disease: A Case-Control Study | Achu Joseph et al., 2023 | India | Case-control | 40 | 18–40 years (Mean: ~23.8) | (1) PCOS+P; (2) PCOS+G; (3) PCOSH; (4) SH (10 per group) | Rotterdam | AAP (1998); Eke et al. (2012) | Medical conditions; medication affecting periodontium (6 mos); obesity |
| Evaluation of oxidative status in patients with chronic periodontitis and polycystic ovary syndrome: A cross-sectional study | Saglam et al., 2018 | Turkey | Cross-sectional | 88 | 19–40 years (Mean: ~28.1) | (1) PCOS+CP (n=22); (2) PCOSH (n=22); (3) SHP (n=22); (4) SHPH (n=22) | Rotterdam | Page & Eke (2007); Flemmig (1999) | BMI ≥ 25 kg/m²; HbA1c ≥ 6.5%; IGT; smoking; alcohol; OCPs, ATB, or insulin-sensitizers (3 mos) |
| Evaluation of periodontal status in different phenotypes of polycystic ovary syndrome in untreated patients of early reproductive age: A case–control study | Işık et al., 2020 | Turkey | Cross-sectional case–control | 206 | 18–24 years (Median: ~21.0) | (1) Phenotype A; (2) Phenotype B; (3) Phenotype C; (4) Phenotype D; (5) SH | Rotterdam | WHO (DMFT Index); PI, GI | Systemic diseases; pregnancy; smoking; alcohol; OCPs, ATB, or steroids (6 mos); periodontal therapy (6 mos) |
| Evaluation of Serum Homocysteine and Nitric Oxide Levels in Women with Polycystic Ovarian Syndrome and Periodontal Diseases | Hameed & Ahmed, 2017 | Iraq | Case-control | 80 | 25–35 years | (1) SG; (2) PCOS+G; (3) SHP; (4) PCOS+CP (n=20 per group) | Rotterdam | AAP (1999); Newbrun (1996) | Smoking; pregnancy; OCPs or hormonal therapy; systemic diseases (DM, HTN); < 20 teeth; ATB/NSAIDs (3 mos) |
| Evaluation of the Salivary Matrix Metalloproteinase-9 in Women With Polycystic Ovaries Syndrome and Gingival Inflammation: A Case-Control Study | Maboudi et al., 2023 | Iran | Case-control | 78 | 16–40 years (Mean: ~26.0) | (1) PCOS+G (n=26); (2) PCOSH (n=26); (3) SH (n=26) | Rotterdam | 2017 WWC | Smoking; DM; malignancy; adrenal/thyroid dysfunction; OCPs (3 mos); <20 teeth; presence of periodontitis |
| Gingival crevicular fluid levels of visfatin in patients with chronic periodontitis and polycystic ovary syndrome | Saljoughi et al., 2020 | Iran | Case-control | 110 | 35+ (Mean: ~45.4) | (1) PCOS+CP (n=30); (2) PCOSH (n=25); (3) SHP (n=23); (4) SHPH (n=32) | Rotterdam | AAP (Advanced CP: CAL ≥ 3 mm, PPD ≥ 5 mm) | Smoking; alcohol; obesity; DM; HTN; systemic infection (6 mos); OCPs, ATB, or antihypertensives |
| Gingival inflammation and leukocyte–endothelium cell interactions in women with polycystic ovary syndrome | Márquez-Arrico et al., 2025a | Spain | Case-control | 104 | 18–45 years (Mean: ~28.3) | (1) SH (n=36); (2) PCOSH (n=44); (3) PCOS+G (n=24) | Rotterdam | 2017 WWC | Inflammatory/autoimmune disease; DM; cancer; bone pathologies; ATB or NSAIDs (6 mos) |
| Gingival Inflammation and Salivary or Serum Granulocyte-Secreted Enzymes in Patients With Polycystic Ovary Syndrome | Akcalı et al., 2017 | Turkey | Cross-sectional | 125 | 17–43 (Mean: 25.7) | (1) PCOSH; (2) PCOSG; (3) SHPH; (4) SG | Rotterdam | 1999 Workshop | BMI > 30 kg/m²; DM; pregnancy; smoking; thyroid/renal/hepatic disorders; OCPs, ATB, or NSAIDs (6 mos) |
| Gingival Inflammation Modulates NLRP3 Inflammasome Signalling in Peripheral Blood Mononuclear Cells of PCOS Patients: A Case-Control Study | Márquez-Arrico et al., 2025b | Spain | Case-control | 104 | 18–45 years (Mean: ~28.3) | (1) SH (n=36); (2) PCOSH (n=44); (3) PCOS+G (n=24) | Rotterdam | 2017 WWC | Inflammatory/autoimmune disease; DM; cancer; bone diseases; ATB or NSAIDs (recent/chronic) |
| Impact of body mass index and polycystic ovary syndrome (PCOS) subtypes on periodontal health in Chinese women with PCOS and periodontitis | Liu et al., 2024 | China | Case-control | 170 | Mean: ~26.6 (Excl. >35) | PCOS+P vs. SHP (matched for age/BMI); Subgroups by BMI and NIH Type I–IV | Rotterdam; NIH Subtypes | 2018 Int. Classification | BMI ≥ 30.0 kg/m²; DM; smoking (≥10 cig/day); pregnancy; OCPs, ATB, or NSAIDs (3 mos); periodontal therapy (6 mos) |
| Influence of polycystic ovary syndrome on the periodontal health of Indian women visiting a secondary health care centre | Varadan et al., 2019 | India | Case-control | 60 | 18–37 years (Mean: ~24.8). | (1) PCOS (n=30); (2) Healthy (n=30) | Rotterdam | PI, mGI, PD, BOP | Thyroid dysfunction; OCPs; systemic disease; tobacco/alcohol; ATB or periodontal therapy (6 mos); <20 teeth |
| Is Interleukin-17 Involved in the Interaction Between Polycystic Ovary Syndrome and Gingival Inflammation? | Özçaka et al., 2013 | Turkey and the United Kingdom | Case-control | 73 | 15–42 years (Median: ~23.5) | (1) PCOSH (n=31); (2) PCOSG (n=30); (3) SHPH (n=12) | Rotterdam | 1999 Workshop; BOP >50% | BMI >30 kg/m²; smoking; DM; HTN; CVD; OCPs, steroids, or insulin-sensitizers |
| Is There an Interaction Between Polycystic Ovary Syndrome and Gingival Inflammation? | Özçaka et al., 2012 | Turkey and the United Kingdom | Cross-sectional case–control | 73 | 20–32.3 years (Median: ~23.5) | (1) PCOSG; (2) PCOSH; (3) SHPH (n=73 total) | Rotterdam | 1999 Workshop; BOP >50% | BMI >30 kg/m²; smoking; alcohol; pregnancy; DM; thyroid/renal/hepatic disorders; OCPs or insulin-sensitizers |
| Periodontal condition and total antioxidant status in serum among patients with polycystic ovarian syndrome | Najafi et al., 2017 | Iran | Cross-sectional | 80 | 20–38 years (Mean: ~29.0) | (1) PCOS (n=40); (2) Infertile Non-PCOS (n=40) | Rotterdam | CPI, PDI; Radiographic bone loss | Smoking; DM; CVD; Cushing syndrome; Adrenal hyperplasia; Thyroid dysfunction |
| Periodontal disease in polycystic ovary syndrome | Dursun et al., 2011 | Turkey | Cross-sectional | 52 | 18–35 (Mean: ~23.5) | (1) Non-obese PCOS (n=25); (2) Healthy Match (n=27) | Rotterdam | Bone loss assessment (Gingivitis vs. Periodontitis) | BMI ≥ 27 kg/m²; smoking; DM/IGT; OCPs or drugs ≥ 3 mos; other endocrine disorders |
| Periodontal status and high-sensitivity C-reactive protein levels in polycystic ovary syndrome with and without medical treatment | Porwal et al., 2014 | India | Cross-sectional | 126 | 15–36 years (Mean: ~23.1) | (1) PCOS-Newly Diagnosed; (2) PCOS-Medical Treatment; (3) SH | Rotterdam | 1999 Workshop; Moderate/Mild CP definitions | Smoking/Alcohol; DM; CVD; Thyroid; ATB (3 mos); Periodontal therapy (6 mos); < 16 teeth |
| Polycystic ovary syndrome is positively correlated with periodontitis: a prospective study | Dangli et al., 2024 | China | Prospective longitudinal study | 60 | 20–34 years | (1) SH (n=15); (2) PCOS (n=28); (3) SHP (n=5); (4) PCOS+P (n=12) | Rotterdam | 2018 AAP/EFP | Systemic disease other than PCOS and periodontitis |
| Salivary Free Testosterone and Gingival Health Condition among a Group of Women with Polycystic Ovary Syndrome. | Tahir & Hussien, 2017 | Iraq | Case-control | 62 | 20–25 years | (1) Newly diagnosed PCOS (n=31); (2) SH (n=31) | Rotterdam | PI, GI (Lӧe & Silness) | Smoking; pregnancy; systemic disease; ATB, NSAIDs, OCPs, or steroids (recent) |
| Salivary High Sensitive C-Reactive Protein and Gingival Health Status among a Group of Women with Polycystic Ovary Syndrome. | Tahir & Hussien, 2019 | Iraq | Case-control | 62 | 20–25 years | (1) PCOS (n=31); (2) SH (n=31) | Rotterdam | PI, GI (Lӧe & Silness) | Smoking; pregnancy; systemic disease; OCPs; ATB or NSAIDs (recent); <20 teeth |
| Subgingival microflora in adolescent females with polycystic ovary syndrome and its association with oral hygiene, gingivitis, and selected metabolic and hormonal parameters | Wendland et al., 2021b | Poland | Cross-sectional, case-control | 55 | 15–19 years (Mean: ~16.4) | (1) Adolescent PCOS (n=32); (2) SH (n=23) | Rotterdam | GI, PI, PD; Gingivitis: BOP ≥ 10% | Systemic disease; hormonal therapy; orthodontic appliances; smoking; ATB (6 mos) |
| Vitamin D Levels and Their Association With Periodontitis in Women With Polycystic Ovary Syndrome: A Cross-sectional Study | Sahithi et al., 2025 | India | Cross-sectional | 100 | 18–40 (Mean: ~33.2) | (1) SHP; (2) PCOS; (3) PCOS+P; (4) SH (n=25 per group) | Rotterdam | Periodontitis: CAL ≥ 3 mm, PD ≥ 5 mm, BOP ≥ 40% | Tobacco; OCPs, ATB, or periodontal therapy (6 mos); DM; malignancy; osteoporosis; pregnancy |
| AAP, American Academy of Periodontology; AAP/EFP, American Academy of Periodontology/European Federation of Periodontology; AES, Androgen Excess Society; AGEs, advanced glycation end products; AOPPs, advanced oxidative protein products; ART, assisted reproductive technology; ATB, antibiotics; BMI, body mass index; BOP, bleeding on probing; CAL, clinical attachment level; CP, chronic periodontitis; CPI, Community Periodontal Index; CVD, cardiovascular disease; DM, diabetes mellitus; DMFT, decayed, missing, and filled teeth; G, gingivitis; GI, Gingival Index; HbA1c, glycated hemoglobin; HTN, hypertension; IGT, impaired glucose tolerance; mGI, Modified Gingival Index; NIH, National Institutes of Health; NSAIDs, non-steroidal anti-inflammatory drugs; OCPs, oral contraceptives; P, periodontitis; PCOS, polycystic ovary syndrome; PCOSG, PCOS with gingivitis; PCOSH, PCOS with healthy periodontium; PD, probing depth; PDI, Periodontal Disease Index; PI, Plaque Index; PPD, probing pocket depth; RCT, randomized controlled trial; SG, systemically healthy with gingivitis; SH, systemically healthy; SHP, systemically healthy with periodontitis; SHPH, systemically and periodontally healthy; SRP, scaling and root planing; WWC, World Workshop Classification. | | | | | | | | | |

**Supplementary Table 3. Summary of the principal salivary biomarkers investigated across inflammatory, oxidative stress, hormonal–metabolic, and microbial mechanistic domains in the included studies.** The table summarizes the reported biomarkers and their proposed mechanistic relevance to the association between polycystic ovary syndrome and periodontal disease. Abbreviations are defined below.

| **Mechanistic Domain** | **Principal Biomarkers Investigated** | **Direction of Association (When PCOS and Periodontal Inflammation Coexist)** | **Number of Supporting Studies** |
| --- | --- | --- | --- |
| Inflammatory (n=17)* | Interleukins & Cytokines (IL-6, TNF-α, IL-17A/F) | ↑ Elevated (Systemic and local baseline) | 10 |
|  | Proteolytic Enzymes (MMP-8, MMP-9) | ↑ Elevated (Exaggerated tissue breakdown) | 5 |
|  | Acute Phase Proteins (hs-CRP) | ↑ Elevated (Systemic inflammatory burden) | 4 |
|  | Endothelial/Adipokine Markers (Endocan, Visfatin, Leptin) | ↑ Elevated (Endothelial activation) | 3 |
| Oxidative Stress (n=10) | Pro-oxidant Products (MDA, 8-OHdG, AGEs, AOPP, NO, MPO) | ↑ Elevated (Amplified oxidative damage) | 8 |
|  | Antioxidant Capacity (TAS, Glutathione, NRF2 pathway) | ↓ Decreased (Compromised defense mechanisms) | 4 |
| Hormonal–Metabolic (n=15) | Hyperandrogenism & Gonadotropins (Total/Free Testosterone, LH, LH/FSH) | ↑ Elevated (Correlates positively with probing depth and inflammation) | 11 |
|  | Insulin Resistance (HOMA-IR, Fasting Insulin) | ↑ Elevated (Parallels severity of subgingival inflammation) | 6 |
|  | Dyslipidemia (Triglycerides, LDL-C) | ↑ Elevated (Shared metabolic risk factors) | 6 |
| Microbial (n=3) | Pathogenic Complex Colonization (P. gingivalis, F. nucleatum, T. forsythia) | ↑ Elevated (Increased subgingival colonization and systemic antibodies in adults) | 2 |
| Abbreviations: 8-OHdG, 8-hydroxy-2'-deoxyguanosine; AGEs, Advanced Glycation End-products; AOPP, Advanced Oxidation Protein Products; F. nucleatum, Fusobacterium nucleatum; FSH, Follicle-Stimulating Hormone; HOMA-IR, Homeostatic Model Assessment for Insulin Resistance; hs-CRP, high-sensitivity C-Reactive Protein; IL, Interleukin; LDL-C, Low-Density Lipoprotein Cholesterol; LH, Luteinizing Hormone; MDA, Malondialdehyde; MMP, Matrix Metalloproteinase; MPO, Myeloperoxidase; NO, Nitric Oxide; NRF2, Nuclear factor erythroid 2-related factor 2; P. gingivalis, Porphyromonas gingivalis; T. forsythia, Tannerella forsythia; TAS, Total Antioxidant Status; TNF-α, Tumor Necrosis Factor-alpha. | | | |

**Supplementary Table 4. Biological and clinical findings reported in the included studies.** The table summarizes periodontal clinical parameters, biological markers, and key study findings evaluating the relationship between polycystic ovary syndrome and periodontal disease. Abbreviations are defined below.

| **Author, Year** | **Sample Source** | **Periodontal Parameters** | **Biomarkers** | **Mechanistic Domain** | **Clinical Findings** | **Direction in PCOS** |
| --- | --- | --- | --- | --- | --- | --- |
| Can Yasar et al. 2025 | Saliva, GCF, Serum | PI, PD, BoP | IL-6, IL-10, ANXA1 | Inflammatory | ↑ BoP in PCOS + Gingivitis group vs systemically healthy-gingivitis group at baseline; the gingivitis groups showed ↑ PI & GCF vs healthy controls; GCF ANXA1 was higher in the PCOS-healthy periodontium group vs systemically healthy-gingivitis group. | ↑ gingival inflammation in PCOS; post-PMPR ANXA1 ↑ in PCOS + gingivitis group but ↓ in systemically healthy-gingivitis group; ANXA1 change associated with E2 & FSH. |
| Helmi et al. 2016 | Serum, Saliva | GI, PI, SFR | Leptin, CRP, and fibrinogen | Inflammatory, Hormonal-Metabolic | GI, PI, and DMFT scores were significantly higher in women with PCOS compared to healthy controls. Conversely, the Salivary Flow Rate (SFR) was significantly lower in the PCOS group. The study found that severe gingival inflammation and destructive periodontitis were significantly correlated with salivary leptin and CRP levels, though the correlation with salivary fibrinogen and SFR was non-significant. | Significant increase in serum and salivary levels of leptin and fibrinogen in women with PCOS vs. Controls. Women with PCOS also showed a significantly higher prevalence of elevated CRP compared to healthy controls, none of whom exhibited high CRP. Decreased salivary flow rate was observed in PCOS patients. Biomarker levels were also found to increase significantly as the number of metabolic syndrome components in PCOS patients increased. |
| Wendland et al. 2021 | Serum, Saliva | PI, GI, PD, BoP | TNF-α, IL-6, and IL-1β, TT, HOMA-IR, Lipid Profile (TG, TC, HDL-C, LDL-C) | Inflammatory, Hormonal-Metabolic | Periodontal parameters (PI, GI, BoP, and PD) did not differ significantly between adolescent girls with PCOS and healthy controls. Gingival health was primarily associated with oral hygiene (PI) and, to a lesser extent, the hormonal and metabolic profile. In the PCOS group, BoP was positively correlated with the insulin resistance index (HOMA-IR). Additionally, salivary testosterone was negatively correlated with GI and BoP in patients with PCOS, suggesting adequate testosterone levels may be necessary for gingival health. | Salivary testosterone and salivary proinflammatory cytokines (TNF-α, IL-1β, and IL-6) were significantly elevated in the PCOS group vs controls. BMI was higher in the PCOS group compared to the control group. All clinical periodontal parameters (PI, GI, BOP%, and PD) were similar between the two groups. |
| Akcalı et al. 2014 | Serum, Saliva | PD, PI, BoP | P. gingivalis, T. forsythia, T. denticola, A. actinomycetemcomitans, P. intermedia, F. nucleatum, S. oralis | Microbial | Clinical periodontal parameters (PI, BOP, and PD) were significantly higher in the gingivitis groups regardless of systemic health status. However, there were no significant differences in these clinical measurements between the gingivitis group with PCOS and the systemically healthy gingivitis group . The study concluded that PCOS may exert a confounding role in gingival inflammation by quantitatively affecting the oral microflora. | Salivary levels of P. gingivalis, F. nucleatum, S. oralis, and T. forsythia were higher in women with PCOS compared to matched systemically healthy women, particularly when gingivitis was present. Serum antibody levels to P. gingivalis, P. intermedia, and S. oralis were elevated in the presence of PCOS. Specifically, in women with PCOS, BOP scores correlated positively and more strongly with the levels of the studied bacterial taxa compared to the systemically healthy group. |
| Zia et al. 2022 | Serum | PI, GI, PPD, CAL | CTX, bone ALP, 25-hydroxyvitamin D, bone mineral density (BMD) | Bone Metabolism & Endocrine Regulation | PCOS + chronic periodontitis patients showed significantly worse periodontal parameters and lower spinal and femoral BMD than healthy controls. Periodontal measures were similar between PCOS + periodontitis and periodontitis-only groups. | Coexisting PCOS and periodontitis were associated with ↑ CTX, ↓ bone ALP, reduced BMD, and higher vitamin D levels compared with healthy controls. |
| Hu et al. 2023 | Serum, Saliva | FMPS, BoP, PD, CAL | IL-6, IL-17A, MMP-8, LH, FSH (LH/FSH ratio), testosterone, prolactin, progesterone, estradiol, triglycerides, total cholesterol, HDL, LDL, fasting plasma glucose, raman spectroscopy molecular signatures | Inflammatory, Hormonal-Metabolic | PCOS patients exhibited significantly greater mean probing depth (PD) compared to non-PCOS participants. Both PD and triglyceride levels were positively correlated with PCOS status. However, in this relatively young cohort, there was no significant difference in the presence of periodontitis between the PCOS and non-PCOS groups. | Significantly higher serum TG and higher serum MMP-8 levels were found in PCOS patients vs Controls. PCOS patients also showed significantly increased LH, T, E2, and LH/FSH ratios. PCOS + Periodontitis (PC-Perio) vs. Periodontitis alone (H-Perio): The mean Raman intensities for salivary hemoglobin, lipids, fatty acids, adenine, and protein were all significantly higher in the group with both conditions. Additionally, serum MMP-8 levels were positively correlated with BOP and PD in the PC-Perio group. |
| Rahiminejad et al. 2015 | Serum | BoP, CAL, PI, PD | FBS, TG, HDL, LDL, Testosterone, NO | Hormonal-Metabolic  Oxidative Stress | Women with PCOS demonstrated significantly higher PI, BoP, and CAL compared to healthy controls. Although a higher percentage of PCOS patients were diagnosed with moderate periodontitis (6.1% vs. 1% in controls), the overall severity of periodontitis and the rate of tooth loss did not differ significantly between the groups. Additionally, when the researchers controlled for confounding factors like PI and BMI, the difference in CAL between groups was no longer statistically significant. | Serum NO, testosterone, and all metabolic markers (FBS, TG, LDL, HDL) showed no significant differences between the PCOS group and the healthy control group. Despite the similarity in systemic biomarkers, the PCOS group exhibited significantly increased clinical signs of periodontal inflammation and destruction (higher PI, BoP, and CAL) compared to healthy controls. |
| Deepti et al. 2017 | Serum | PI, GI, PD, CAL, BoP | hsCRP, HOMA-IR, fasting blood sugar (FBS), fasting insulin (FI), lipid profile (TG, Cholesterol, LDL, VLDL, HDL), FSH, LH, testosterone, prolactin. | Inflammatory, Hormonal-Metabolic | Non-surgical periodontal therapy (SRP) combined with myo-inositol (MI) reduced systemic inflammation (hsCRP) more than MI alone. MI improved hormonal and metabolic markers (testosterone, LH/FSH ratio, HOMA-IR) in both groups, while adjunct periodontal therapy significantly improved all periodontal clinical parameters. | Women with PCOS and chronic periodontitis had significantly higher hsCRP levels and worse periodontal clinical scores (PI, GI, BoP, PD, CAL) than systemically and periodontally healthy controls. After treatment with Myo-inositol, metabolic and hormonal markers (FSH, LH, Testosterone, FI, and HOMA-IR) in PCOS patients became comparable (no significant difference) to healthy controls. However, even after successful periodontal therapy, hsCRP and periodontal parameters remained significantly higher in the PCOS group compared to the healthy group. hsCRP was found to have a positive correlation with nearly all periodontal and metabolic parameters, and HOMA-IR was significantly correlated with all periodontal parameters. |
| Akcalı et al. 2015 | Serum, Saliva | PD, PI, BoP | MMP-8, TIMP-1, MMP-8/TIMP-1 ratio | Inflammatory | ↑ PI, BOP, and PD in gingivitis groups compared to the periodontally healthy groups; no significant differences between the gingivitis patients with or without PCOS. | ↑ salivary MMP-8, ↑ serum MMP-8, ↑ MMP-8/TIMP-1 ratio; Elevated serum MMP-8 observed irrespective of gingival status. Positive correlation between periodontal indices (PD, BOP, PI) and MMP-8 levels is present only in PCOS. |
| Saglam et al. 2020 | Serum, Saliva | PI, GI, PD, CAL, BoP | Endocan, IL-6 | Inflammatory | ↑ PI, GI, PD, CAL, and BOP% in periodontitis groups compared to healthy controls. BOP% was significantly higher in PCOS with periodontitis (PCOSP) than in periodontitis alone, suggesting enhanced gingival bleeding in PCOS. | ↑ Serum endocan (all PCOS groups), ↑ salivary endocan (PCOS + periodontitis), ↑ salivary IL-6 (PCOS + periodontitis vs PCOS healthy); Inflammatory biomarker elevation was most pronounced when PCOS and periodontitis coexisted. |
| Damodaran et al. 2025 | Saliva | BOP, PPD, CAL, PISA | Advanced Glycation End products (AGEs) | Oxidative Stress | PCOS with periodontitis (PCOS+P) group showed the highest salivary AGEs and the poorest periodontal parameters (OHI-S, BOP, PPD, CAL, PISA). PISA positively correlated with salivary AGEs across all groups. PCOS patients with healthy periodontium (PCOS+H) exhibited higher BOP% and PISA than healthy controls, suggesting PCOS contributes to inflammatory burden independent of periodontal disease. | ↑ salivary AGEs, ↑ gingival bleeding (BOP%), ↑ inflamed periodontal surface area (PISA), ↑ PPD & CAL when periodontitis is present |
| Dharuman et al. 2022 | Serum, Saliva | PD, CAL, BoP, PISA | Advanced Oxidation Protein Products (AOPP). | Oxidative Stress | The PCOS with periodontitis (PCOSCP) group exhibited the greatest probing depth and the highest PISA values compared to all other groups. Women with PCOS also demonstrated higher PISA scores than healthy controls, indicating that PCOS independently contributes to gingival inflammation even in the absence of periodontitis. | ↑ salivary & serum AOPP, ↑ PISA ,↑ BOP |
| Joseph et al. (2023) | Serum, Saliva | PPD, CAL, BoP, PI, PISA, PESA | Fusobacterium nucleatum, Porphyromonas gingivalis | Microbial | ↑ BOP, PPD, and CAL in the PCOS with periodontitis group compared to others. PESA and PISA values differed significantly among all groups, with PCOS patients presenting with gingivitis or periodontitis showing higher PISA scores than healthy controls, indicating increased systemic inflammatory burden. | ↑ Fusobacterium nucleatum (ascending prevalence from healthy → PCOS → PCOS + periodontitis), ↑ Porphyromonas gingivalis (elevated in PCOS with gingival inflammation; not statistically significant), ↑ BOP, PPD, CAL, PISA |
| Saglam et al. 2018 | Serum, Saliva | PD, CAL, BoP, GI, PI | 8-OHdG, MDA, TAS | Oxidative Stress | PD, CAL, GI, BOP, and PI were significantly higher in chronic periodontitis (CP) groups compared to periodontally healthy controls. No significant differences in clinical periodontal parameters were observed between CP patients with PCOS and systemically healthy CP patients. Salivary 8-OHdG and MDA levels were significantly elevated in CP groups versus healthy controls. | ↑ Serum 8-OHdG, ↑ Serum MDA, ↓ Serum TAS (total antioxidant status), ↑ Highest oxidative stress levels when PCOS and CP coexist |
| Işık et al. 2020 | Serum | PD, CAL, BoP, GI, PI | tT, fT, LH, FSH, Estradiol, DHEA-S, 17-OHP, Prolactin, TSH, Insulin, Glucose, HOMA-IR, Triglycerides, Total Cholesterol, LDL-C, HDL-C | Hormonal-Metabolic | Probing depth (PD) was significantly higher in all phenotypic subgroups of PCOS compared to the healthy control group. No significant differences were found in other periodontal parameters, including PI, GI, BoP, CAL, or DMFT index, between women with PCOS and healthy volunteers. | ↑ FT and LH levels in the total PCOS group compared to the control group. ↑TC & LDL-C in Phenotype B (Hyperandrogenism + Ovulatory Dysfunction) compared to controls. No significant difference in Fasting Glucose, Fasting Insulin, or HOMA-IR levels between PCOS groups and the healthy controls. |
| Hameed, D. J., & Ahmed, M. A. 2017 | Serum | PI, GI, BoP, PPD, CAL | Hcy, NO | Oxidative Stress | Women with PCOS and gingivitis (Group 2) exhibited the highest mean values for GI and BOP. The group with both PCOS and chronic periodontitis (Group 4) showed the highest mean values for PPD and CAL. While the Plaque Index (PLI) was highest in the group with gingivitis alone, clinical periodontal destruction was consistently more severe when PCOS was present. | Both serum Hcy and NO levels were highest in the PCOS + chronic periodontitis group. PCOS patients with gingivitis had significantly higher Hcy and NO levels than those with only gingivitis. Women with both PCOS and chronic periodontitis had significantly higher Hcy and NO levels than women with chronic periodontitis who were systemically healthy. |
| Maboudi et al., 2023 | Saliva | GI, PI, BoP | MMP-9 | Inflammatory | All gingival indices (GI, PI, and BOP) were significantly higher in women with PCOS and gingivitis compared to women in the other two groups. Additionally, while results for all groups were within normal reference ranges, women with PCOS and gingivitis had significantly higher salivary MMP-9 levels than all other groups. | Increased salivary MMP-9 levels in women with PCOS, regardless of their gingival status, compared to healthy controls. Increased scores for clinical indicators of gingival inflammation (GI, PI, and BOP) in women with PCOS compared to systemically healthy controls. |
| Saljoughi et al., 2020 | GCF | BoP, CAL, PPD | Visfatin | Inflammatory, Hormonal–metabolic | Clinical periodontal parameters were significantly worse in the groups with periodontitis. The PCOS-CP group (PCOS with advanced chronic periodontitis) exhibited a mean PPD of 6.86 ± 0.91 mm and CAL of 3.92 ± 0.98 mm, which were significantly higher than the healthy control group. Additionally, BoP was significantly elevated in both periodontitis groups compared to healthy individuals. | Increased GCF visfatin levels were observed in all disease groups compared to healthy controls. The PCOS-CP group had the highest visfatin levels compared to all other groups. Visfatin levels were significantly higher in women with both conditions than in those with only advanced CP. Women with PCOS but a healthy periodontium showed higher mean visfatin levels than the healthy control group. |
| Márquez-Arrico et al., 2025 | Venous blood | BOP, CAL, PPD  PI, CI | hsCRP, TNFα, MPO, Glutathione, JNK expression, sP-selectin, neutrophil–endothelium interactions (rolling velocity, rolling flux, cellular adhesion) | Inflammatory, Oxidative Stress, Hormonal–metabolic | PCOS patients with gingivitis (PCOS+) exhibited significantly higher bleeding on probing (BOP), plaque index, and calculus index compared to both healthy controls and PCOS patients without gingivitis. In contrast, no significant differences were observed between groups for PPD, CAL, or the number of teeth lost, indicating the periodontal disease in this cohort was primarily gingival inflammation rather than advanced tissue destruction. | Increased systemic inflammatory markers (hsCRP, TNFα) and increased oxidative stress (MPO) were found in all PCOS patients compared to controls, regardless of gingival status. Decreased levels of the antioxidant glutathione were also noted in PCOS. Neutrophils in women with PCOS showed increased JNK expression and increased sP-selectin. PCOS patients exhibited decreased rolling velocity and increased rolling flux and cellular adhesion. These subclinical atherosclerotic markers were more pronounced in PCOS patients with gingivitis, suggesting that gingival inflammation acts synergistically with the syndrome to exacerbate the risk of cardiovascular disease. |
| Akcalı et al., 2017 | Serum, Saliva | PD, PI, BoP | MMP-9, MPO, NE, MMP-9/TIMP-1 ratio | Inflammatory | Clinical parameters (PI, BOP, and PD) were significantly higher in the gingivitis groups compared to periodontally healthy groups, regardless of systemic status. However, there were no significant differences in these clinical periodontal measurements when comparing women with PCOS to systemically healthy controls within the same periodontal diagnostic category. | Lower salivary MMP-9 was observed in periodontally healthy women with PCOS compared to healthy controls. Conversely, serum MMP-9 and MPO levels were significantly increased in women with both PCOS and gingivitis (Pg) compared to those with PCOS and a healthy periodontium (Ph) or systemically healthy women with gingivitis (Hg). In the presence of PCOS, there was a significant positive correlation between clinical periodontal parameters (PD, BOP, PI) and serum MMP-9, salivary MPO, NE, and the MMP-9/TIMP-1 ratio. This contrasts with systemically healthy women, who showed a negative correlation between periodontal parameters and serum MMP-9. |
| Márquez-Arrico et al., 2025 | Peripheral Blood Mononuclear Cells (PBMCs), Serum | BoP, CAL, PPD, PI | NFκB p65, JNK, NLRP3, ASC, procaspase-1, caspase-1, total superoxide, NRF2, GCLC, GSR, hsCRP, TNFα, serum IL-1β | Inflammatory, Oxidative Stress | The PCOS with gingivitis (PCOS+) group exhibited significantly higher BOP, plaque index, and calculus index compared to both healthy controls and women with PCOS but no periodontal disease. There were no significant differences found between the groups regarding probing pocket depth (PPD), clinical attachment level (CAL), or tooth loss. | Women with PCOS (regardless of gingival status) showed increased total superoxide and an impaired antioxidant response (significantly lower mRNA levels of NRF2, GCLC, and GSR). They also showed increased systemic inflammation (higher hsCRP and TNFα). The presence of gingivitis in PCOS patients led to a significant increase in protein levels of NFκB p65, JNK, NLRP3, ASC, procaspase-1, and caspase-1, suggesting that gingival inflammation modulates and exacerbates the systemic innate immune response. |
| Liu et al., 2024. | Venous blood | PD, GBI | PRL, FSH, LH, E2, AD, T | Hormonal–metabolic | Women with PCOS and periodontitis exhibited a significantly higher GBI compared to systemically healthy controls with periodontitis. However, PD and the percentage of deep periodontal pockets were similar between the two groups. The study also found that GBI differed significantly across different PCOS subtypes and that a higher BMI further intensified gingival inflammation. | Significant variation in AD levels was observed across different PCOS subtypes. No significant difference was found in PD or the levels of PRL, LH, FSH, E2, and T across the PCOS subtypes in this study. |
| Varadan et al., 2018. | Serum, GCF | PI, mGI, PD, BoP | MDA, MPO, Free Testosterone, FBS, Insulin, Triglycerides, HOMA-IR | Oxidative Stress, Hormonal–metabolic | Women with PCOS exhibited significantly higher PI, mGI, and BOP scores compared to healthy controls. While most participants were in a younger age group with shallow pockets (PD < 6 mm), 7 of the 30 PCOS patients had deeper pockets (6 mm). | Significant increases in the PCOS group for serum and GCF MDA, free testosterone, FBS, and TG. No significant difference was found for MPO (serum/GCF) or fasting insulin levels. There was a moderate positive correlation between Serum MDA and clinical inflammation. |
| Özcxaka et al., 2013 | GCF, Saliva, Serum | PD, PI, BoP | IL-17A, IL-17F, IL-17A/F (heterodimer), IL-17E (IL-25), Insulin, Cortisol, DHEAS, Testosterone, Progesterone | Inflammatory, Hormonal–metabolic | The PCOS + gingivitis group exhibited significantly greater PI, PD, and GCF volumes compared to the healthy PCOS group and healthy controls. Serum IL-17A and IL-17F correlated positively with the Ferriman-Gallwey score (FGS) and PD. | IL-17A, IL-17F, and IL-17A/F were significantly increased in serum and GCF/saliva in women with PCOS compared to healthy controls. Serum IL-17A and IL-17F correlated positively with FGS and Probing Depth. IL-17E was significantly decreased in serum and GCF in the PCOS groups, particularly in those with gingivitis. Serum IL-17E showed a negative correlation with PD, BOP, and PI. Interestingly, salivary IL-17E levels were higher in the PCOS groups than in controls. |
| Özcxaka et al., 2012 | GCF, Saliva, Serum | PD, PI, BoP | TNF-α, TNF-αR1, TNF-αR2, IL-6, Insulin, Cortisol, Free Testosterone, 17-OH Progesterone, DHEAS | Inflammatory | The PCOS + gingivitis group exhibited significantly higher GCF, saliva, and serum IL-6 concentrations than the PCOS + healthy group. Additionally, insulin, cortisol, and free testosterone were significantly higher in the PCOS + gingivitis group compared to the PCOS + healthy group. Clinical periodontal parameters (PI, BOP, and PD) showed strong positive correlations with serum and GCF IL-6 levels. | ↑ Salivary TNF-α, TNF-αR1, and TNF-αR2 in all women with PCOS compared to healthy controls. ↑ Serum IL-6 and TNF-α levels were found in both PCOS groups compared to controls, with the highest levels observed when PCOS and gingivitis coexisted. Increased systemic markers of hyperandrogenism (Free Testosterone, DHEAS) and increased insulin were present in women with PCOS compared to controls. |
| Najafi et al., 2017 | Serum | CPI, PDI | TAS | Oxidative Stress | Bone loss was significantly more prevalent in the PCOS group compared to the control group. The PCOS group also demonstrated significantly higher mean PDI scores and a higher frequency of advanced CPI scores compared to controls. | No significant difference in serum TAS between PCOS patients and controls. However, across the entire study population, higher TAS levels were significantly associated with lower (better) CPI scores, suggesting a link between antioxidant capacity and periodontal health. |
| Dursun et al., 2011. | Serum, GCF | PI, GI, BoP, PD, CAL | MPO, NO | Oxidative Stress | PCOS patients exhibited significantly higher PD, GI, BoP, and PI. GCF volume was also higher in the PCOS group. No periodontal bone loss was detected, indicating the disease was at the gingival level (gingivitis). | ↑ GCF MPO and GCF NO in the PCOS group compared to controls. No significant difference was found in serum NO levels between the groups. |
| Porwal et al., 2014 | Serum | PI, GI, BoP, PD, CAL | hs-CRP | Inflammatory | Women newly diagnosed with PCOS (PCOS-N) exhibited significantly higher BOP, PD, and CAL, as well as a higher prevalence of moderate periodontitis, compared to both healthy controls and those receiving medical treatment. Conversely, women on medical treatment (PCOS-MT) for at least 6 months showed significantly lower measures of periodontal breakdown and systemic inflammation compared to the newly diagnosed group. | ↑ Serum hsCRP levels in newly diagnosed PCOS patients compared to controls. Positive correlation between systemic inflammation (hsCRP) and local periodontal destruction (BOP and CAL). Medical treatment for PCOS is associated with a decrease in hsCRP and a reduction in periodontal inflammation. |
| Hu et al. 2024 | Serum, Saliva | FMPS, BoP, PD, CAL | IL-6, IL-17A, TNF-α, MMP-8, LH, FSH, LH/FSH ratio, total testosterone, prolactin, progesterone, estradiol | Hormonal–Metabolic, Inflammatory | PCOS and periodontitis are positively correlated, with their progression accompanied by changes in pro-inflammatory cytokines and sex hormones. LH and the LH/FSH ratio were positively correlated with PD. Additionally, salivary MMP-8 levels were positively correlated with all clinical periodontal parameters (PD, BOP, FMPS, and CAL site rates). While PCOS patients did not always have higher periodontitis prevalence in this young cohort, those with both conditions (Group D) exhibited significantly higher periodontal destruction. | Increased serum MMP-8 levels in PCOS patients compared to healthy controls. Increased salivary MMP-8 specifically in the PCOS + periodontitis group compared to the PCOS-only group. Increased LH and LH/FSH ratio in PCOS groups (with or without periodontitis) compared to healthy controls. Increased serum IL-6 levels in PCOS patients compared to non-PCOS subjects over a 6-month follow-up. |
| Tahir & Hussien, 2017 | Saliva | Pl, GI | Salivary free Testosterone | Hormonal–metabolic | GI was significantly higher in women with PCOS compared to the control group. Interestingly, PI was significantly lower in the PCOS group than in the control group, which the authors attributed to possible oral health negligence or lack of motivation in the control group. No significant correlation was found between the levels of salivary free testosterone and the clinical oral variables (PI and GI). | Salivary free testosterone levels were significantly higher in women with PCOS compared to healthy controls. Gingival inflammation was increased in women with PCOS despite them having lower plaque scores than the control group. Statistical analysis indicated that salivary free testosterone has high sensitivity and specificity (100% for both) as a diagnostic marker for PCOS. |
| Tahir & Hussien, 2019 | Saliva | Pl , GI | hs-CRP | Inflammatory | Gingival inflammation (GI) was significantly higher in women with PCOS compared to the control group, despite the PCOS group having a significantly lower Plaque Index (PlI). | ↑ Salivary hs-CRP levels in women with PCOS compared to healthy controls. In PCOS group, hs-CRP levels correlated positively and significantly with the GI, but not with the PI. |
| Wendland et al., 2021 | Subgingival plaque, fasting blood samples | PI, GI, PD, BoP | 9 periodontal pathogens (including P. gingivalis, T. denticola, T. forsythia, and A. actinomycetemcomitans), FSH, LH, total/free testosterone, estradiol, DHEA-S, SHBG, fasting glucose/insulin, lipid profile (TC, HDL-C, LDL-C, TG). | Microbial, Hormonal-Metabolic | No significant differences between adolescent females with PCOS and healthy controls regarding periodontal clinical parameters (PLI, GI, BOP%, and PD) or the total subgingival bacterial count. Only six participants (three in each group) exhibited moderate gingivitis. | No increase in pathogenicity: Contrary to expectations, PCOS was not associated with higher subgingival pathogenicity; the control group actually had significantly higher levels of P. micros and a greater frequency of infection by T. denticola and orange complex bacteria. PCOS subjects had significantly higher BMI and increased insulin resistance (HOMA-IR). C. gingivalis count was positively correlated with estradiol, while HDL-C levels were negatively correlated with A. actinomycetemcomitans and orange complex bacteria. |
| Durga Sahithi et al., 2025 | Serum | PD, CAL, BoP | 25-hydroxyvitamin D, Total Testosterone, Total Cholesterol, Triglycerides, HDL, LDL, FBS | Hormonal–metabolic | ↑ PD and Cal in the groups with periodontitis compared to the periodontally healthy groups. No significant difference in these parameters between women with PCOS and systemically healthy controls within the same periodontal diagnostic category. | No significant differences of serum Vitamin D levels between women with PCOS and healthy controls. Women with PCOS exhibited increased total testosterone, increased triglycerides, and increased LDL compared to healthy controls. Conversely, HDL levels were significantly decreased in women with PCOS compared to healthy participants. Fasting blood sugar levels showed no significant variation across the study groups. |
| Abbreviations: AL = Attachment Level; BOP = Bleeding on Probing; CAL = Clinical Attachment Level; CPI = Community Periodontal Index; FMPS = Full Mouth Plaque Score; GBI = Gingival Bleeding Index; GI = Gingival Index; mGI = modified Gingival Index; PD/PPD = Probing (Pocket) Depth; PI = Plaque Index; PISA = Periodontal Inflamed Surface Area; PESA = Periodontal Epithelial Surface Area; SFR = Salivary Flow Rate; AD = Androstenedione; DHEA-S = Dehydroepiandrosterone sulfate; E2 = Estradiol; FBS/FPG = Fasting Blood Sugar/Fasting Plasma Glucose; FI = Fasting Insulin; FSH = Follicle-Stimulating Hormone; fT/TT = free/Total Testosterone; HDL-C = High-Density Lipoprotein Cholesterol; HOMA-IR = Homeostatic Model Assessment for Insulin Resistance; LDL-C = Low-Density Lipoprotein Cholesterol; LH = Luteinizing Hormone; OHP = Hydroxyprogesterone; PRL/TSH = Prolactin/Thyroid- Stimulating Hormone; SHBG = Sex Hormone-Binding Globulin; TC = Total Cholesterol; TG = Triglycerides; VLDL = Very-Low-Density Lipoprotein; 8-OHdG = 8-Hydroxy-2&amp;#39;- deoxyguanosine; AGEs = Advanced Glycation End products; ALP = Alkaline Phosphatase; ANXA1 = Annexin A1; AOPP = Advanced Oxidation Protein Products; ASC = Apoptosis- associated speck-like protein; CRP/hs-CRP = (high-sensitivity) C-Reactive Protein; CTX = C-terminal telopeptide; GCLC/GSR = Glutamate-cysteine ligase catalytic subunit/Glutathione reductase; Hcy = Homocysteine; IL = Interleukin (e.g., IL-1B, 6, 10, 17A, 17F, 25); JNK = c-Jun N-terminal kinase; MDA = Malondialdehyde; MMP = Matrix Metalloproteinase (e.g., MMP- 8, 9); MPO = Myeloperoxidase; NE = Neutrophil Elastase; NFkB = Nuclear Factor kappa B; NO = Nitric Oxide; NRF2 = Nuclear factor erythroid 2-related factor 2; NLRP3 = NLR family pyrin domain containing 3; TAS = Total Antioxidant Status; TIMP-1 = Tissue Inhibitor of Metalloproteinases-1; TNF-alpha = Tumor Necrosis Factor-alpha; A. actinomycetemcomitans = Aggregatibacter actinomycetemcomitans; F. nucleatum = Fusobacterium nucleatum; P. gingivalis = Porphyromonas gingivalis; P. intermedia = Prevotella intermedia; S. oralis = Streptococcus oralis; T. denticola = Treponema denticola; T. forsythia = Tannerella forsythia. | | | | | | |
